# Spiral resonators for on-chip laser frequency stabilization

**DOI:** 10.1038/ncomms3468

**Published:** 2013-09-17

**Authors:** Hansuek Lee, Myoung-Gyun Suh, Tong Chen, Jiang Li, Scott A. Diddams, Kerry J. Vahala

**Affiliations:** 1 T.J. Watson Laboratory of Applied Physics, California Institute of Technology, Pasadena, California 91125, USA; 2 hQphotonics, Pasadena, California 91106, USA; 3 Time and Frequency Division, National Institute of Standards and Technology, Boulder, Colorado 80305, USA

## Abstract

Frequency references are indispensable to radio, microwave and time keeping systems, with far reaching applications in navigation, communication, remote sensing and basic science. Over the past decade, there has been an optical revolution in time keeping and microwave generation that promises to ultimately impact all of these areas. Indeed, the most precise clocks and lowest noise microwave signals are now based on a laser with short-term stability derived from a reference cavity. In spite of the tremendous progress, these systems remain essentially laboratory devices and there is interest in their miniaturization, even towards on-chip systems. Here we describe a chip-based optical reference cavity that uses spatial averaging of thermorefractive noise to enhance resonator stability. Stabilized fibre lasers exhibit relative Allan deviation of 3.9 × 10^−13^ at 400 μs averaging time and an effective linewidth <100 Hz by achieving over 26 dB of phase-noise reduction.

## Abstract (web summary)

Optical reference cavities are important in precision time keeping and low-noise microwave generation. Here as a step towards their miniaturization, the authors demonstrate a chip-based reference cavity that uses a spiral geometry to improve stability by introducing thermal and mechanical immunity.

## Graphical abstract

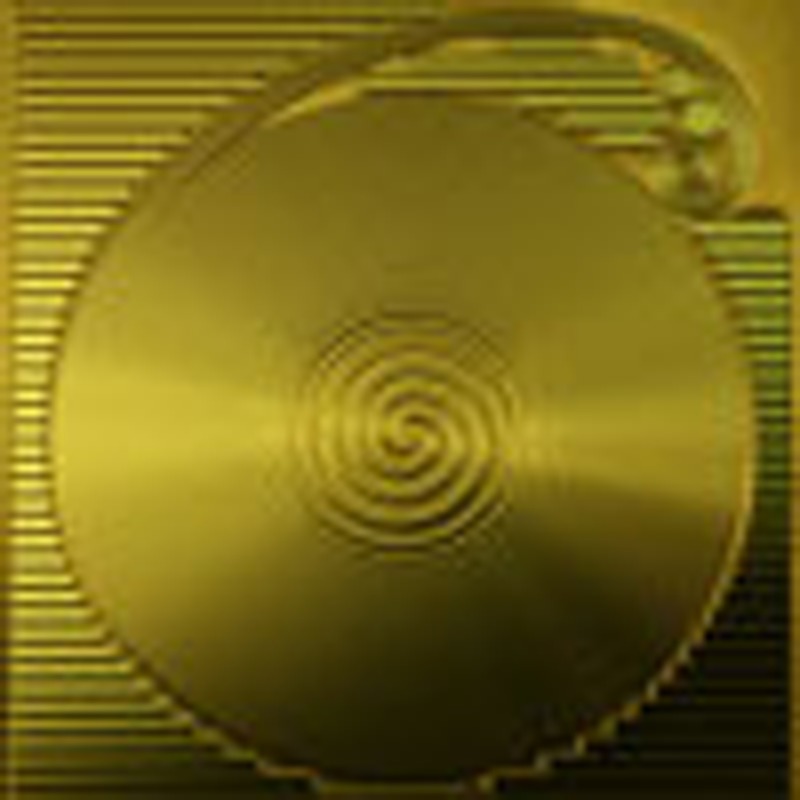

Applications including gravity-wave detection^1^, optical clocks^2^ and high-performance microwave generation^3^ have fuelled interest in frequency references for stabilization of laser sources. Such references benefit from high optical *Q* factor or equivalently long optical storage time and systems in-use or under investigation include Fabry–Perot cavities^4^,^5^,^6^, absorption spectral-hole burning in cryogenically cooled crystals^7^,^8^ and long-delay-line interferometers^1^,^9^. Bench-top-scale systems based on Fabry–Perot optical cavities have attained an Allan deviation 1 × 10^−16^ at 1 s averaging^5^. In these systems, high-finesse-mirrors create a narrow resonance for laser locking, whereas low-thermal-expansion housings and low-thermal noise mirror coatings create immunity to thermal fluctuations that perturb the resonant frequency^4^,^5^,^6^,^10^,^11^. With the advent of ultra-high optical-*Q*, solid-state resonator systems based on silica^12^,^13^,^14^ and crystalline fluoride materials^15^,^16^,^17^ attention has naturally turned towards miniature devices. Besides their compact size, these devices, through their reduced mass, can offer improved performance with respect to shock and acceleration. In the case of chip-based devices, there is also the potential for integration with other components.

In solid-state resonators, fluctuations arise from thermorefractive, thermo-mechanical, elasto-optic and photo-thermal noise^18^,^19^,^20^. The first three mechanisms are fundamental, while the fourth is determined by the transfer of the laser power fluctuations into thermal changes of the cavity refractive index and size. Crystalline resonators are advantageous for reduced thermorefractive noise as the dependence of refractive index on temperature is low in comparison with silica^19^. Along these lines, locking of a laser to a MgF_2_ resonator has attained stabilization to 6 × 10^−14^ at 0.1 s averaging time^21^. It has also been demonstrated that dual-mode feedback control can be used to stabilize the absolute frequency of a resonator by measuring modal temperature using two, orthogonally polarized modes^22^,^23^,^24^. Moreover, application of these resonators as frequency stabilization elements in ring fibre lasers^25^,^26^ and designs for enhanced acceleration and vibration immunity have been proposed^27^,^28^.

In this paper, we study the application of a chip-based, high-*Q* resonator in the form of a spiral for laser frequency stabilization. Besides being the first chip-based reference cavities, the geometry offers a high level of immunity to thermorefractive noise, as well as thermo-mechanical and photo-thermal noise. Also, the measurements are performed without any special vacuum isolation and temperature control. Phase-noise spectra are measured and show strong laser phase-noise suppression over offset frequencies ranging from 1 Hz to 100 kHz. Frequency fluctuations, as characterized by the Allan deviation, are also measured.

## Results

### Design of low-thermal noise reference cavities

In the absence of resonator noise sources, the rms frequency difference of a locked laser relative to a cavity line center depends upon the optical *Q* and signal-to-noise ratio (SNR) of the detected laser signal through the following expression^29^,


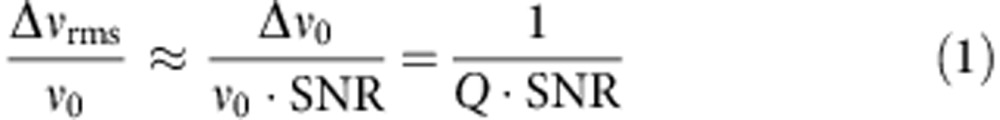


where *Q*=*ν*_0_/Δ*ν*_0_ has been used in the result from the study by Drever *et al*.^29^ and where the SNR depends upon the integration time or servo-control bandwidth. Given a high enough *Q* factor and large enough SNR, the stability of the laser locked to the cavity becomes determined by fluctuations in the cavity line centre, itself. Excluding technical noise sources such as acceleration and acoustics, the largest contribution to these fluctuations originates from thermorefractive noise^18^,^19^. Intuitively, if one considers N, randomly dispersed fluctuators that each contribute an rms frequency fluctuation *σ*_1_, then the total rms frequency fluctuation will scale like 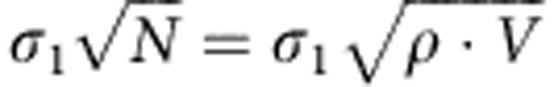 (assuming the fluctuators are uncorrelated) where *ρ* is the density of fluctuators and *V* is the mode volume. At the same time, if the fluctuators have a fixed size, then the coupling of the fluctuators to the mode will diminish as the mode volume increases and *σ*_1_ will vary like 1/*V*. Therefore, the total rms frequency fluctuation will scale like 
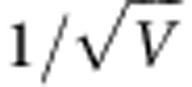
. This scaling is apparent in models of thermal-related fluctuations in resonators^18^,^19^. In optical fibre reference cavity systems, this source of noise is reduced by employing long fibre delays^9^.

**Figure 1:**
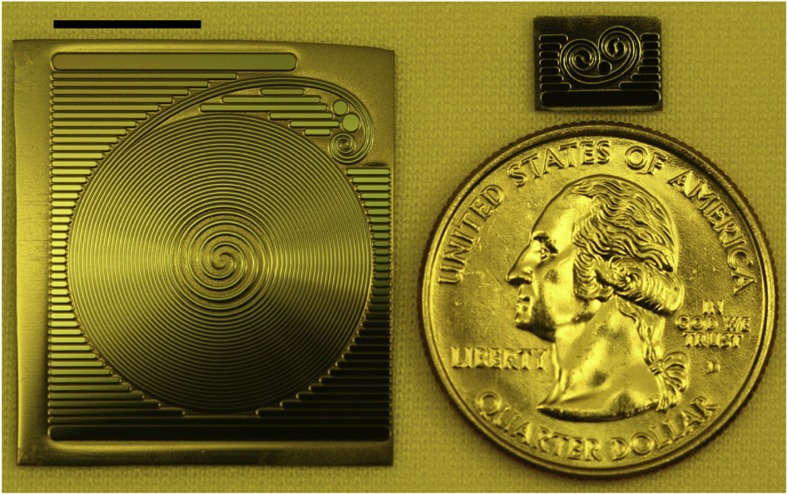
Photograph of spiral waveguide resonators. Left: 1.2 m spiral resonator. Upper-right: 4.5 cm spiral resonator used for studies of *Q* scaling. Lower right: quarter shown to provide scale. Scale bar, 1 cm.

To leverage this scaling on a silicon chip, a resonator in the form of a spiral is used (see Fig. 1). Spiral resonators have previously been used to create narrow free-spectral range devices on a chip^30^. To attain both high-*Q* and large-mode volume a special ultra-low-loss waveguide is used in the current work, providing optical waveguide loss as low as 0.037 dB m^−1^ (refs 14, 31). Using these waveguides we demonstrate resonators that are over 1 m in length with *Q* factors in excess of 100 million, but for which the device footprint is smaller than 5.4 cm^2^. Beyond the thermorefractive noise immunity offered by these devices, they also provide enhanced immunity to photo-thermal noise. This happens because of their large mode volume, which greatly reduces the circulating intensity at a given coupled optical power and hence also the tendency for heating of the mode volume. As a result, it is possible to obtain high SNR (see equation. (1)) without degradation in resonator stability. Moreover, thermo-mechanical noise is greatly suppressed as this form of noise varies inverse-quadratically with resonator length^32^.

**Figure 2:**
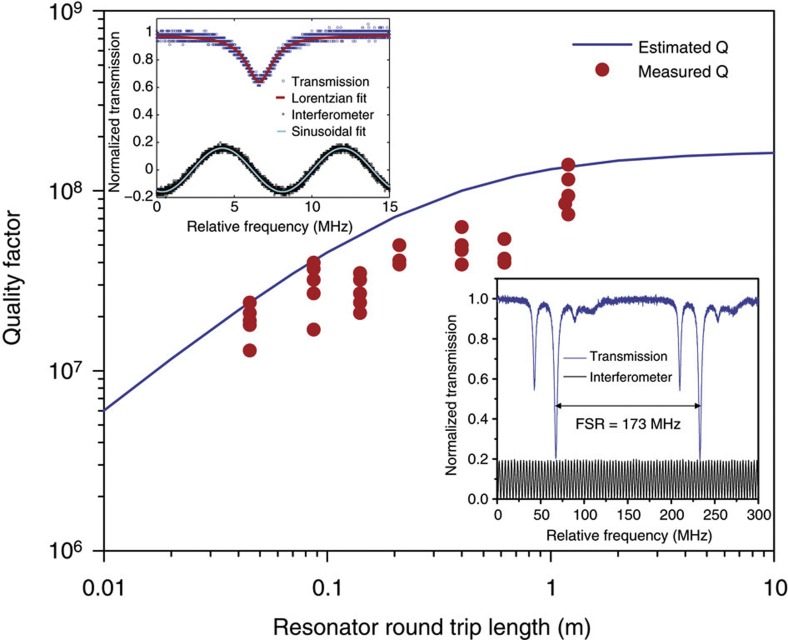
Intrinsic *Q* factor measured for the various resonator lengths. The maximum *Q* factor obtained was 140 million at a length of 1.2 m. The blue curve is a theoretical prediction for the *Q* versus length that assumes a waveguide loss of 0.15 dB m^−1^. Upper left: a typical optical spectrum in blue. The sinusoidal curve is an interferometer scan that is used to calibrate the linewidth. Lower right: spectral scan in excess of one free-spectral range for the TE polarization. The black curve is the interferometer calibration trace.

Representative long and short, round-trip path-length resonators are shown in Fig. 1. The resonators contain two interleaved spiral waveguides with *S*-turn adiabatic couplers at the spiral centre. Details on the process used to fabricate the waveguides are described in previous work^31^. Optical coupling to the resonators occurrs in the upper-right corner of the chip and uses a fibre taper^33^,^34^. *Q* measurements are performed by monitoring transmitted optical power on the taper coupler while scanning an external cavity semiconductor laser across a free-spectral range (FSR) of the resonator. The lower right inset of Fig. 2 shows a typical scan in the longest resonator having a round-trip physical path length of 120 cm. The measured FSR of the device (173 MHz) agrees well with the expected FSR based on the round-trip length. The other resonances seen in the inset correspond to higher-order transverse modes. The spectrum is remarkably uncluttered. We attribute this to spatial filtering of higher-order transverse modes by the adiabatic couplers (*S*-bend waveguide turns at the centre of each spiral)^35^. To verify the dependence of *Q* factor on resonator length a range of device lengths are tested (4.5, 8.7, 14, 21, 40, 62 and 120 cm). The results are plotted in the main panel of Fig. 2 along with a theoretical estimate of the *Q* factor based upon an adiabatic coupler loss of 0.02 dB per coupler and a waveguide loss of 0.15 dB m^−1^. The agreement is reasonable. Also, the waveguide loss here is higher than reported in earlier work on account of using a contact aligner for micro-fabrication as opposed to a projection (stepper) lithography system^14^,^31^. Nonetheless, a maximum *Q* factor of 140 million is obtained.

### Phase-noise spectrum and linewidth

**Figure 3:**
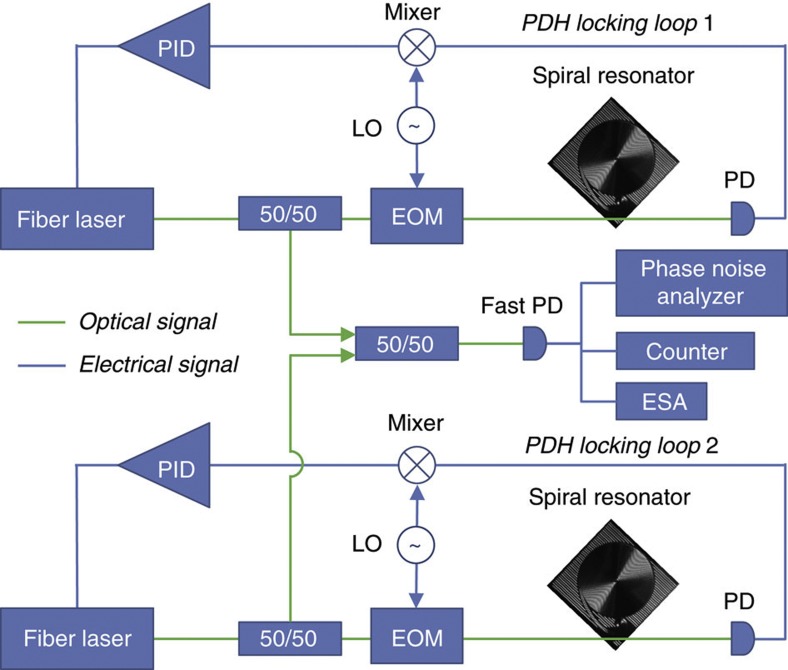
Experimental setup. Two, fibre lasers independently locked to high-*Q* spiral reference cavities using Pound–Drever–Hall (PDH) locking systems. Each PDH locking loop includes a photodiode (PD), electro-optic modulator (EOM), local oscillator (LO) and proportional-integral-differential feedback controller (PID). The outputs of the separately locked lasers were combined on a photodetector and the resulting photocurrent was analysed using an electrical spectrum analyser (ESA), a frequency counter and a phase-noise analyser. All components are on the same optical table.

To measure the frequency stability of the spiral resonators, the experimental setup shown in Fig. 3 was used. It includes two, fibre lasers (Orbits Lightwave, at optical frequencies near 193.43 THz) that are locked with separate Pound–Drever–Hall systems^29^ to two, high-*Q*, silica-on-silicon spiral resonators. In the measurements, ~3 mW of laser power is in the fibre-taper waveguide and about 1 mW of this power is coupled into the resonator. The locked fibre lasers were heterodyned to produce a beat signal near 350 MHz. This beat note directly reveals the combined phase-noise of the two stabilized lasers, and it was analysed using an electrical spectrum analyser, a phase-noise analyser (Rohde & Schwarz FSUP26) and a frequency counter (Tektronix FCA3120 and Pendulum CNT-91). Acoustic shielding was placed around the entire setup to attenuate environmental sound; also, pumps and instrumentation in adjoining rooms were turned-off during measurements. Measurements were performed for several cases: free-running fibre lasers, lasers locked to the 1.2 m spiral reference cavities, and, for comparison purposes, lasers locked to independent conventional disk resonators of varying diameters (3, 7.5 and 15 mm)^14^. In prior work, linear drift has been substracted from data^9^,^21^. In the present work, no linear drift correction was performed.

**Figure 4:**
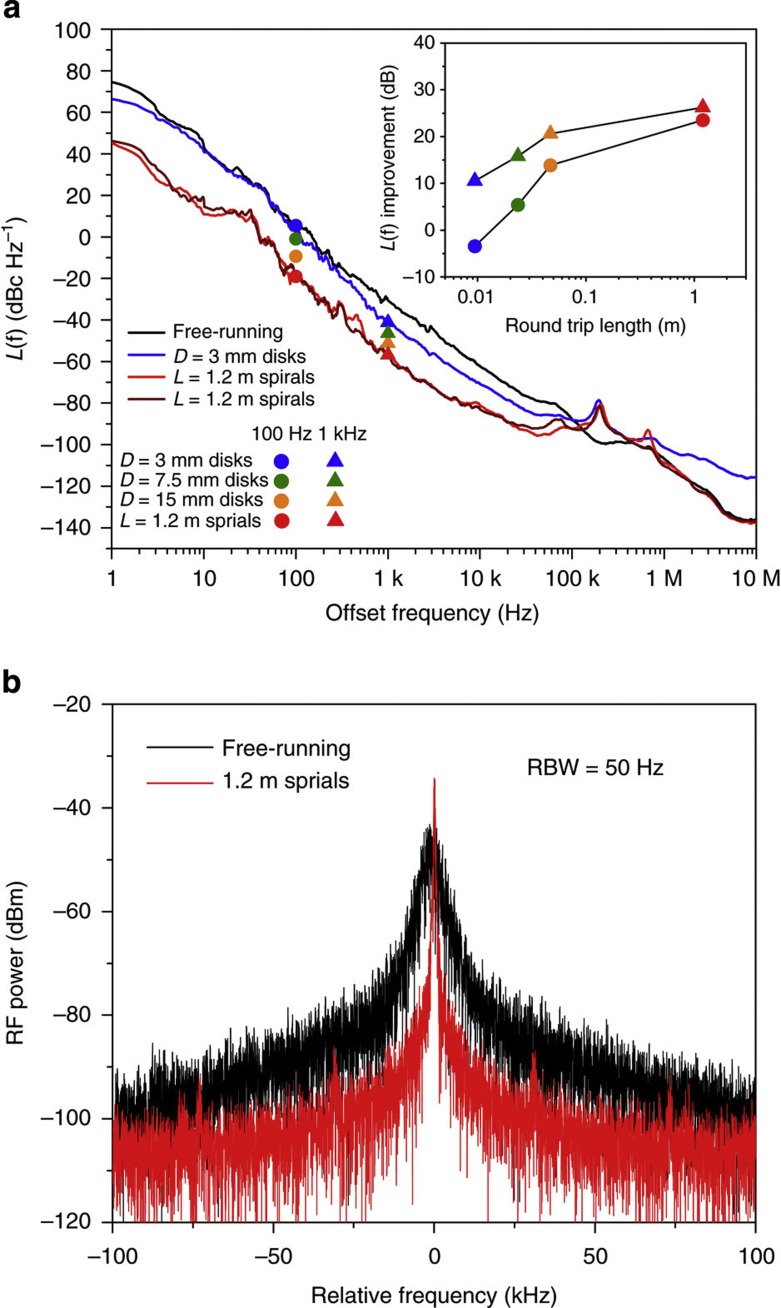
Phase-noise spectra and beat-note spectral measurement. (**a**) Phase-noise spectra measured for two, free-running 193 THz fibre lasers (black), fibre lasers independently locked to two, 3 mm disk resonators (blue), and fibre lasers independently locked to two, 1.2 m long spiral resonators (red). To test measurement reproducibility, resonators were characterized on multiple days. Measurement of the 1.2 m long resonators on a second day is shown as the dark-red trace. The data for the spiral resonator show an average suppression by 26 dB of the fibre laser noise when locked to the spiral resonators. In comparison, 10 dB of noise suppression is observed using the 3 mm device. The servo-control noise bumps appear at around 200 kHz for the locked phase-noise spectra. The inset is a plot of the noise suppression at 100 Hz and 1 kHz offset frequencies plotted versus resonator length for each of the resonators tested. (**b**) The electrical spectrum of the fibre lasers’ beat note for both free-running and locked configurations. Spectral narrowing and noise suppression are apparent in the locked spectrum.

Figure 4a shows the phase-noise spectral density function for the heterodyned signals both with and without the locking systems engaged. Data are shown for the free-running lasers, 3 mm disk resonators and the 1.2 m spiral cases. The spectra were measured over offset frequencies from 1 Hz to 10 MHz and an instrument smoothing algorithm has been applied to show the trend. Within the bandwidth of the feedback control system (bandwidth limit is delineated by the servo-control bumps visible near 200 kHz in the phase-noise spectrum) an average of 26 dB suppression of fibre-laser phase-noise was measured when the fibre lasers were locked to the 1.2 m spirals. In comparison, only 10 dB of suppression was achieved with the 3 mm diameter disks (measured at 1 kHz offset frequency). Below 1 kHz offset frequency even better suppression was observed for spiral locking versus disk locking. We believe this is caused by better immunity to photo-thermal noise in the spiral resonators on account of their larger mode volume. For example, the 3 mm disk, phase-noise spectrum degrades at offset frequencies less than 1 kHz, which is consistent with the thermal corner frequency observed in other silica-based resonators^36^. In the inset of Fig. 4a, the noise suppression improvement relative to the free-running fibre laser case is plotted for each of the resonators measured. The data here are taken at 1 kHz offset and also at 100 Hz offset to illustrate the improved suppression of noise at lower offset frequency provided by the spiral resonator. Overall, there is roughly a 1/*f*^3^ dependence of phase-noise on frequency. This is indicative of flicker noise and the dependence is consistent with resonator modelling of thermorefractive noise, which generally feature a roll-off in frequency that is faster than 1/*f*^2^ down to low-offset frequencies^18^,^19^.

Figure 4b shows a comparison of the measured electrical spectrum generated upon heterodyne detection with the free-running fibre lasers to the case when the lasers are independently locked to the 1.2 m long resonators. For this measurement, the resolution bandwidth (RBW) of the electrical spectrum analyser was set to 50 Hz, resulting in an 80 ms sweep time over the 200 kHz span. As an additional comparison, we have calculated the effective linewidths for the beat note of the two, stabilized lasers from the phase-noise spectra^37^ and found 900 Hz (free-running lasers), 400 Hz (locked to 3 mm disks) and 100 Hz (locked to 1.2 m spiral resonators). The individual laser linewidths will be narrower than these values. The calculated, beatnote linewidth of the lasers locked to 1.2 m spiral resonators is consistent with both the electrical spectrum measurement of the beatnote as well as the Allan deviation measurement result.

### Allan deviation measurement

**Figure 5:**
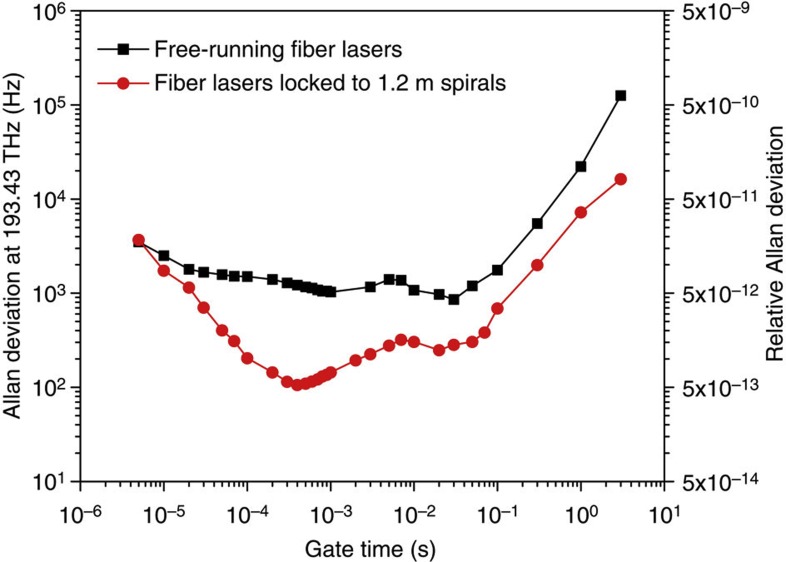
Allan deviation measurement result. Allan deviation of the beat frequency between the two, free-running fibre lasers (black squares), and for the lasers independently locked to two, 1.2 m long spiral resonators (red circles) is shown. A minimum Allan deviation of 100 Hz at an optical frequency of 193 THz, corresponding to a relative Allan deviation of 5.5 × 10^−13^, was measured at a gate time of 400 μs for 10 dB improvement compared with the free-running case. Assuming the fibre lasers are independent and equivalent, a relative Allan deviation of 3.9 × 10^−13^ is expected for each locked laser.

In order to further confirm the frequency stabilization by the spiral resonators, Allan deviation measurements^38^ were carried out using a Tektronix FCA3120 frequency counter (Fig. 5). As an additional check the measurements were also confirmed using a Pendulum CNT-91 frequency counter. Zero dead-time measurements were performed using both frequency counters. Over the range of gate times from 5 μs to 3 s, Allan deviations of the spiral-locking case were improved in comparison with the free-running (unlocked) case. At a gate time of 400 μs, a minimum relative Allan deviation of 5.5 × 10^−13^ was measured, which is ten times lower than that of the free-running case. If the two lasers are assumed to be independent, then the relative deviation of a single, stabilized laser is 3.9 × 10^−13^, or an equivalent Allan deviation of 75 Hz. After this minimum there is a rise, then decrease and final rise in the Allan deviation. The resulting local maximum near 10 ms is believed to result from environmental fluctuations and to also be associated with the corresponding increase around 10–60 Hz in the phase-noise spectrum in Fig. 4a. It was also noteworthy that stability improvement of locked signal at longer gate times is consistent with the phase-noise suppression at low-offset frequency (<10 Hz).

### Mechanically induced noise

Measurements of thermo-mechanical-induced noise were also conducted using both the disk and spiral resonators. The optomechanical coupling parameter is expected to vary inversely with cavity length so that phase-noise exhibits an inverse quadratic dependence on length^32^. This dependence was observed over a range of cavity lengths by using the Hänsch Couillard technique^39^,^40^. Spectral features believed to be thermally excited mechanical resonances were observed at offset frequencies greater than 1 MHz, and steadily diminished in amplitude to levels below the sensitivity limit of the system for the largest spirals measured (1.2 m path length). As confirmed in Fig. 4a, there was no evidence of mechanical noise in the phase-noise spectra measurements.

## Discussion

It is noteworthy that ideal frequency division of the 193 THz optical carrier to 10 MHz would provide a signal with close-to-carrier phase-noise of ~−100/*f*^3^ dBc Hz^−1^. This is a level that is already competitive with the state-of-the-art oven-controlled crystal oscillators, and the basic architecture to accomplish this has been demonstrated with the combination of laboratory frequency combs and electronic division^41^. It is intriguing to consider that the spiral cavity demonstrated here could be the frequency reference for a chip-integrated platform, that together with advances in microcomb technology^42^,^43^ would ultimately provide broad-bandwidth synthesis of low-phase-noise signals from the optical to the RF. In addition, the ability to reduce the effective linewidth of a fibre laser by a factor of 10X using only a chip-based device is of practical importance in any applications requiring high coherence. This includes coherent fibre-optic communications^44^,^45^, remote sensing^46^ and atomic physics^4^,^47^. Moreover, aside from simple acoustical shielding of the experimental setup and operation on a floated optical table, there has been no attempt to thermally stabilize or vibration isolate these devices. Likewise, there has been no drift correction of the data. Concerning future performance improvements, optical-fibre-based reference systems using 1 km fibre delays have attained a phase-noise level of −83 dBc Hz^−1^ at 1 kHz offset frequency^9^. In the current chip-based design, 27 metre long delay lines have been demonstrated and lengths in excess of 100 m are feasible^31^. Finally, thicker oxides may be possible if thermal oxidation is replaced by processes such as the flame hydrolysis method. The combination of these methods could produce a 1,000-fold increase in mode volume relative to the current results.

## Author contributions

H.L., T.C. and K.J.V. conceived the devices and all authors helped to design the experiment. H.L. fabricated the devices with assistance from T.C. M.G.S. measured the devices with assistance from the other authors. All authors helped to write the paper.

## Additional information

**How to cite this article:** Lee, H. *et al*. Spiral resonators for on-chip laser frequency stabilization. *Nat. Commun.* 4:2468 doi: 10.1038/ncomms3468 (2013).
